# Supplementary material for: Definer: A computational method for accurate identification of RNA pseudouridine sites based on deep learning
Source: PLoS One. 2025 Apr 24;20(4):e0320077. doi: 10.1371/journal.pone.0320077 (PMC12021131; doi:10.1371/journal.pone.0320077)
Supplement: Attachment — Submitted filename: PONE-D-24-30581_reviewed_manuscript.docx [file pone.0320077.s001.docx]

The manuscript titled "**Definer-PseU: A computational method for accurate identification of RNA pseudouridine sites based on deep learning**” developed the **Definer-PseU** software, which predicts pseudouridine sites in RNA sequences using deep learning methods. Pseudouridine plays an essential role in the stability and function of RNA. The authors used datasets from three different species: *Homo sapiens*, *Saccharomyces cerevisiae*, and *Mus musculus*. The authors have combined three different deep learning models—the convolutional neural network (CNN), the gated recurrent unit (GRU), and the attention mechanism for the predictions. They showed improved prediction as compared to existing prediction methods. Below are some minor comments/suggestions that, if addressed, could significantly enhance the manuscript.

1) Small correction in the Introduction section
   a. at line three "**RNA modifications is the pseudouridine ()**": please fill the bracket as per required symbol
   b. In line five, Author mentioned the “**mRNA as an example of non-coding RNA”**, but mRNA is a coding RNA.
2) In Figure 1, kindly correct the word "**Date collection**" to "Data collection" and "**Date**" to "Data".
3) The author explained the different nucleotide distribution patterns between pseudouridine and non-pseudouridine sites using the Two Sample Logo webserver in Figure 4. However, it's unclear how they distinguished between pseudouridine and non-pseudouridine sites and they did not mention the statistical test used for this analysis. Additionally, Figure 4 does not present all nucleotide sequences, is there any specific reasons for this.
4) Author has unnecessarily explained methods like "One hot ecncoding", CNN method etc. These are not needed and could be removed.

For example: "let is say we have a car's color attribute, with possible values of "red", "blue", and "green". Using One-hot encoding, we can convert this attribute into a binary array of three elements. For instance, if the car's color is "blue", the encoded result would be [0,1,0], where the second element is 1, indicating that the car is blue. In some machine learning models, using raw categorical variables may cause problems such as in linear regression and logistic regression. One-hot encoding can solve this problem by converting categorical variables into equivalent numeric data, allowing the model to process these variables correctly. It is important to note that when there are many possible values for the categorical variable, using One-hot encoding can lead to a sharp increase in data dimensions, which may affect the efficiency and accuracy of the model. Therefore, in practical applications, it is necessary to decide whether to use One-hot encoding and how to encode it based on specific circumstances."

5) In section 2.4.2 CNN: Author explained the working model of CNN, which does not need to be explained and can be easily removed. However, Author can explain more about hyperparameters in the CNN model e.g. how many convolutional layers/max pooling layers they are using in their model, any padding they used, what is the size of the kernel/filter? why did they use only ReLU activation function in CNN? Did they use activation function at each convolving/max pooling operation, which max pooling did they use and why, how many epochs did they use during the training of the data?
6) Authors have many common/spelling mistakes which can be corrected before final publication. However, this is not primary objective but it would be great, if this error will be fixed before the final publication for better read.
   a. Add a space before the opening square bracket, for example, "among others [5, 6]."
   b. "data collecting," can be change to data collection.
   c. The scientific name should be written in full (e.g., *Homo sapiens*) on its first occurrence, followed by the abbreviation (e.g., *H. sapiens*) in subsequent mentions and be consistent.

**Note:** These minor errors are found throughout the manuscript; above are just few guided examples.
