# Supplementary material for: Definer: A computational method for accurate identification of RNA pseudouridine sites based on deep learning
Source: PLoS One. 2025 Apr 24;20(4):e0320077. doi: 10.1371/journal.pone.0320077 (PMC12021131; doi:10.1371/journal.pone.0320077)
Supplement: Attachment — Submitted filename: review cover.docx [file pone.0320077.s003.docx]

January 9, 2025

Dear Editor-in-Chief,

We would like to express our sincere gratitude for your constructive feedback and the opportunity to revise our manuscript. We have carefully considered all the comments from the reviewers and the academic editor, and have made significant efforts to address each point.

Response to the Concern about the Software Figure

We acknowledge the concern regarding the appearance of the software figure. The figure was indeed not a simple screenshot. In fact, it is a visual representation of our in - house developed visualization software. This software is designed to provide users with an intuitive interface to interact with the model's outputs.

To clarify this, we have made the following improvements:

In the revised manuscript, we have added a detailed description in the figure caption explaining that it is a visual of our dedicated visualization software. We have also included a sentence stating that the software can be downloaded and run from [https://github.com/Mumika/Definer-Software-engineering]. This not only addresses the concern about the figure's appearance but also provides a clear way for interested readers to access and experience the software themselves.

We'll conduct a thorough proofreading to correct all spelling, formatting mistakes and ensure consistent use of scientific names as per your examples.

Thank you again for your time and guidance.

Sincerely,

XinFeng, Ph.D.

Email: [fengxin@jlict.edu.cn](mailto:fengxin@jlict.edu.cn)

School of Science, Jilin Institute of Chemical Technology
